# ICL Postimplantation Decentration and Tilt in Myopic Patients with Primary Iridociliary Cysts

**DOI:** 10.1155/2023/3475468

**Published:** 2023-01-16

**Authors:** Ying Wang, Ruibo Yang, Yue Huang, Chen Zhang, Hui Liu, Zhe Jia, Shaozhen Zhao

**Affiliations:** Tianjin Key Laboratory of Retinal Functions and Diseases, Tianjin Branch of National Clinical Research Center for Ocular Disease, Eye Institute and School of Optometry, Tianjin Medical University Eye Hospital, Tianjin 300384, China

## Abstract

**Purpose:**

To observe the decentration and tilt of implantable collamer lens (ICL) as well as possible visual effects postimplantation in primary iridociliary cysts.

**Methods:**

The present investigation was a retrospective cohort study. All 48 patients (91 eyes) who underwent ICL surgery at the Center of Refraction Surgery of Tianjin Medical University Eye Hospital between July 2018 and May 2020 were split into two groups based on the absence or presence of primary iridociliary cysts established by ultrasonic biological microscopy (UBM) examination. Intraocular pressure (IOP), corrected distance visual acuity (CDVA), uncorrected distance visual acuity (UDVA), anterior chamber angle (ACA), anterior chamber volume (ACV), and anterior chamber depth (ACD) were recorded preoperatively and postoperatively at 1, 6, and 12 months. Additionally, we performed an analysis of the ICL vault, decentration, and tilt using a rotating Scheimpflug Oculus Pentacam camera system at 1, 6, and 12 months after surgery.

**Results:**

No serious complications were observed. Significant postoperative improvement (*P* < 0.05) of UDVA was established in the two studied groups; however, we did not observe statistically significant intergroup differences (*P* > 0.05) throughout the entire research period. In each group, the preoperative ACA, ACV, and ACD were statistically significantly reduced (*P* < 0.05), but no such decrease was established between their postoperative values (*P* > 0.05). We observed no statistical differences between both groups with regard to their values of IOP, ACA, ACV, ACD, ICL vault, ICL decentration, and tilt at 1, 6, and 12 months after surgery. Similarly, no statistically significant within-group correlation (*P* > 0.05) of the decentration of ICL and the tilt and the CDVA values was established.

**Conclusion:**

No postimplantation effect of ICL with a central hole on vision was established in myopia patients with primary iridociliary cysts, within certain limits of ICL decentration and tilt values.

## 1. Introduction

Myopia and astigmatism corrections have extensively been performed by the use of the implantable collamer lens (ICL). It is noteworthy that the use of ICL that features a central 0.36 mm hole (EVO-ICL) has eliminated the necessity for iridotomy and has allowed the maintenance of an appropriate aqueous flow, which is important for normal physiological processes in the anterior segment. EVO-ICL implant has been largely demonstrated to be an efficient and safe method of myopia correction [1–4].

**Figure 1:**
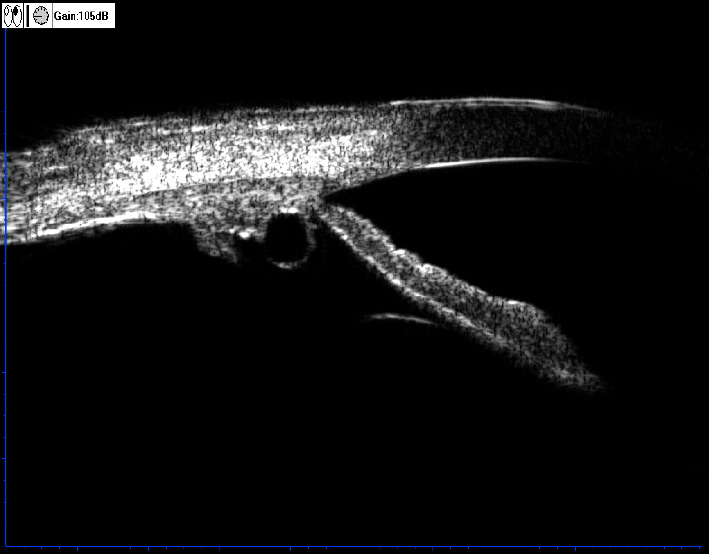
UBM images of one patient with a ciliary cyst.

Primary iridociliary cysts are frequently occurring benign uveal tumors. In a previous study, the reported primary iridociliary cyst incidence ranged from 4.9% to 64.3% in the general population [5] but was more common in high-myopia patients. The majority of primary iridociliary cysts are shaded by the iris and can be found by ultrasonic biological microscopy (UBM). A cyst is characterized as solitary or multiple, round or ovoid, with a thin and hyperechoic wall and a sonolucent or hypoechoic interior (Figure 1).

Theoretically, ciliary cysts could be in contact with ciliary sulcus-implanted ICL. Thus, it is logical to assume that iridociliary cysts would influence the ICL implantation location. Furthermore, the progressive development of such ciliary sulcus cysts can induce distortion in the anatomy of the anterior chamber. Additionally, a haptic shift can be caused by cysts, and ICL might be prevented from stretching, elevating the vault, and causing ICL decentration and tilt and even pupillary block and/or glaucoma. Another adverse effect could be exerted by a large number of cysts located within the toric ICL (TICL) axis, which can lead to rotation disruption and result in astigmatism and glare and negatively affect visual acuity. Gharaibeh et al. [6] described a case, in which the intraocular lens placement was affected by a hidden iris cyst, causing glaucoma. Zeng et al. [7] reported a dislocation of the ICL caused by a cyst.

Our study aimed to elucidate the influence that a primary iridociliary cyst could exert in terms of postoperative EVO-ICL decentration and tilt. We performed measurements of the preoperative and postoperative values of the intraocular pressure (IOP), visual acuity, anterior chamber angle (ACA), anterior chamber volume (ACV), anterior chamber depth (ACD), as well as of the postoperative ICL vault, decentration, and tilt of participants with and without primary iridociliary cysts who underwent EVO-ICL implantation. The purpose of our research study was to assess whether iridociliary cysts had an impact on ICL decentration and tilt. We also aimed to determine whether decentration and tilt of the ICL were correlated with visual acuity.

## 2. Patients and Methods

### 2.1. Patients

In the present retrospective study, we included patients with EVO-ICL implantation performed between July 2018 and May 2020 at Tianjin Medical University Eye Hospital. The total number of enrolled patients was 48 (91 eyes), of which 13 were male (26 eyes) and 35 were female patients (65 eyes). We divided the patients into the following two groups. The cyst group consisted of a number of 47 eyes with primary ciliary bodies and/or iris cysts detected by UBM. The noncyst group included 44 eyes without cysts. To be recruited, the patients had to meet certain inclusion criteria, which are as follows: corrected distance visual acuity (CDVA) ≥10/20; stable refraction power for over two years (with a myopia increase ≤−0.5 *D* every year); the endothelial cell density (ECD) had to be more than 2000 cells/mm^2^; and the value of ACD had to be 2.80 mm or higher. The participants with eye diseases (such as cataracts and glaucoma) and systemic diseases (connective tissue disease and diabetes mellitus.) affecting the vision were excluded.

All investigators followed the principles of the Helsinki Declaration 2008, and Tianjin Medical University Eye Hospital's ethical committee approved the protocol (2021KY(L)-15).

### 2.2. Data Collection and Image Analysis

Before surgery, the patients were subjected to a comprehensive ophthalmic evaluation, including CDVA, uncorrected distance visual acuity (UDVA), as well as standard slit-lamp microscopic, funduscopic, IOP (Canon, Japan), white-to-white (WTW), ECD (Topcon, Japan), and UBM (SW-3200, SUOER, China). Central corneal thickness (CCT) and axial length (AL) were determined by LENSTAR (Haag-Streit, Switzerland). Measurements of ACA, ACV, ACD, the vault of the ICL, the decentration of the ICL, and the tilt of the ICL were performed using Pentacam eye examination (Oculus Instruments, Germany). The follow-up periods covered 12 months. In 1, 6, and 12 months postoperatively, we observed the changes in the CDVA, UDVA, IOP, ACA, ACV, ACD, and ICL vault, as well as the decentration and tilt of the ICL. All examinations were completed by the specialist with full expertise.

In the darkroom, the patients were asked to watch the indicator light continuously. During that time, the Pentacam visual segment analysis system was used to obtain the ICL 360-degree full-circle Scheimpflug image by its built-in rotating Scheimpflug camera. Another built-in camera in the center of the Oculus Pentacam system was employed to detect pupil size and direction and maintain the control of eye fixation. When a good focus of the eye was achieved, 25 images which marked “OK” in the imaging quality specifications (QSs) window were obtained per eye, and the horizontal and vertical meridians were selected. The brightness and contrast of the images were enhanced to improve the clarity of the ICL anterior and posterior curve images for the acquisition of vault and tilt quantitative measurement data, with the maximum tilt value as the final result of the eye. The clearest image of the ICL central hole was selected for quantitative measurement data acquisition of the decentration.

**Figure 2:**
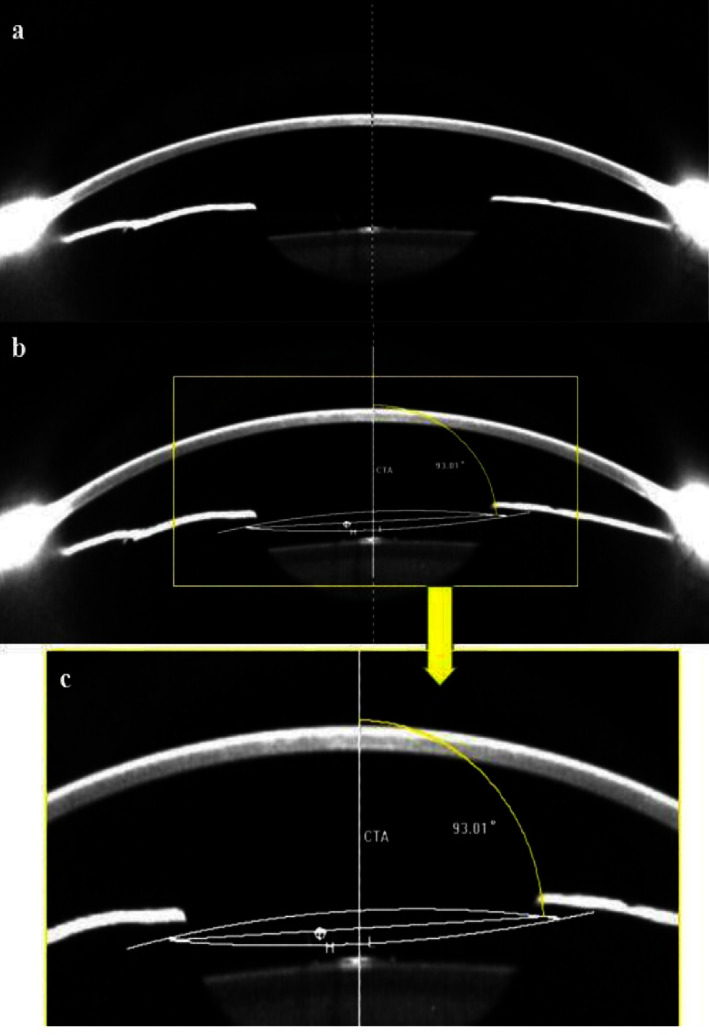
The anterior segment pentacam image with the ICL horizontal. (a) The white point *H* on the processed image (b) indicates the ICL central hole, while line *L* represents the plane of the ICL; the ICL axis runs perpendicular to the ICL plane. (c) The ICL tilt degree (between the CTA and the ICL axis) is 93.01°–90° = 3.01°.

As can be seen in Figure 2(a), we obtained a high-quality anterior segment image, in which the ICL was located in the horizontal plane. The ICL's central hole was determined by the most intensive reflective point on its inner surface. The vertical dotted line on this image is the corneal topographic axis (CTA), detected by Pentacam, which links the corneal vertex to the fixation point on the corneal topographer. In this study, we defined the ICL vault as being the height between the posterior curve of the ICL and the anterior curve of the crystalline lens on the CTA. Then, we calculated the ICL decentration using the perpendicular distance between the CTA to the ICL's center hole. The ICL central vault and decentration values were measured on the diagram of the Pentacam overview with the software program for image analysis of the device. We recorded the mean values of three measurements.

Furthermore, we adjusted two arcs to fit the ICL posterior and anterior curvatures with the ImagePro Plus 6.0 software. In this image, the intersecting arcs crossed the ICL plane segment. The line perpendicular to the ICL plane indicated the ICL axis. The angle between the ICL plane and CTA represented tilt, which was calculated as 90° and was subtracted from the value of the angle between the plane of the ICL and the CTA (Figure 2).

### 2.3. Surgical Procedure

At surgery initiation, we inserted an ICL into the anterior chamber via a 3.0 mm corneal incision utilizing a STAAR Surgical injector. After a regular amount of viscoelastic surgical agent (Amvisc; Bausch & Lomb, Shandong, China) was administered over the ICL, the ICL was adjusted into the posterior chamber, and we flushed the viscoelastic surgical agent out of the eye with a balanced salt solution. Postoperatively, we prescribed combined treatment with steroidal and antibacterial eye drops, as well as artificial tears. The same experienced surgeons (SZZ) performed all surgical operations.

### 2.4. Statistical Analysis

SPSS 25.0 (IBM Corporation, USA) was used for the statistical analysis of the data. For our statistical analysis, CDVA and UDVA conversion were implemented to the logMAR scale. We adopted the Kolmogorov F02D Smirnov test to validate normal data distribution. In addition, we used nonparametric and parametric tests for comparisons of continuous variables, based on the specific distribution of data. The independent *t*-test or Mann–Whitney *U* test was conducted for continuous variables analysis. In contrast, the Fisher exact probability or the chi-square tests were implemented to compare categorical variables and conduct evaluations of the intergroup differences. The IOP, ACA, ACV, ACD, vault, decentration, and tilt of the ICL were evaluated using the multivariate analysis of variance (MANOVA) for repeated measures. Tukey's posthoc multiple comparison tests were used for pairwise comparisons. Spearman correlation analysis was performed to establish the correlation between CDVA and the ICL decentration and tilt. Statistical significance was determined at a *P* value of less than 0.05.

## 3. Results

In this retrospective study, we included a total number of 48 patients (91 eyes). The baseline clinical preoperative and demographic indicators of the participants are listed in Table 1. In the intergroup comparison, no statistical differences were observed in the sex ratio, age, manifest spherical equivalent, CDVA, UDVA, IOP, AL, ACA, ACV, ACD, WTW, CCT, and ECD (*P* > 0.05).

### 3.1. Visual Outcomes

Importantly, all of the performed implantations were successful, without postoperative complications throughout the entire follow-up period. No patients' postoperative CDVA declined, and no significant between-group difference was observed during the follow-up study (*P* > 0.05). We found statistically significant improvements in both groups' postoperative UDVA (*P* < 0.05); however, we detected no statistically significant between-group difference throughout the entire follow-up durations.

### 3.2. IOP, ACA, ACV, and ACD

**Figure 3:**
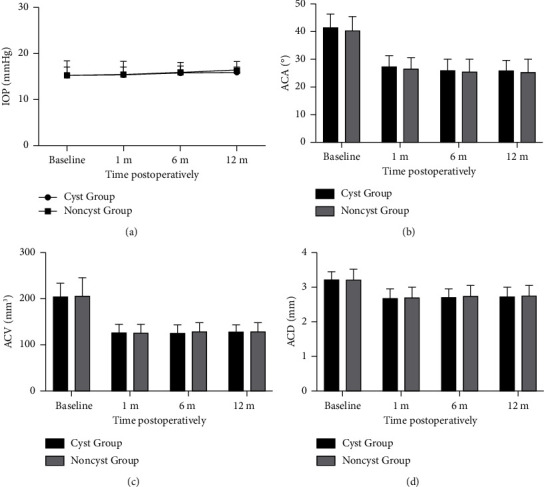
Mean IOP (a), ACA (b), ACV (c), and ACD (d) of the cyst group and the noncyst group at each follow-up at different time points. ^*∗*^*P* < 0.05, the error bar indicates the standard deviation.

We found no statistical differences within the groups in the mean preoperative and postoperative (1, 6, and 12 months following surgery) IOP values (*P* > 0.05). A significant decline was noted in the preoperative values of ACA, ACV, and ACD (*P* < 0.05) in the two studied groups; however, no such differences were detected between the postoperative values (*P* > 0.05). Additionally, we established no statistical differences between-groups in their values of IOP, ACA, ACV, and ACD at the 1-, 6-, and 12-month postoperative follow-up durations (Figure 3).

### 3.3. Vault, Decentration, and Tilt

**Figure 4:**
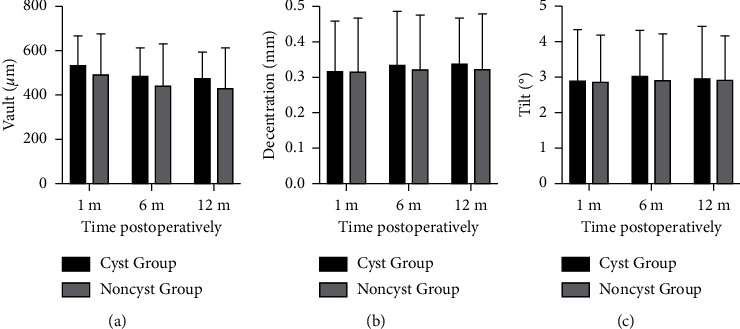
Mean vault (a), decentration (b), and tilt (c) of the cyst group and the noncyst group at each follow-up at different time points. ^*∗*^*P* < 0.05, the error bar indicates the standard deviation.

No statistically significant within-group differences were established in either of the groups in the vault, decentration, and tilt at the different time points of the follow-up examination (*P* > 0.05). The vault, decentration, and tilt values showed no statistical differences in postoperative follow-up examinations (Figure 4).

### 3.4. Correlation between Postoperative ICL Decentration and Tilt and Visual Acuity

The values of CDVA and the ICL decentration and tilt are listed in Table 2. We established no statistical correlation between the degree of ICL decentration and tilt and CDVA.

## 4. Discussion

The application of implantable ICL is effective and safe for myopia and astigmatism. EVO-ICL's central port promotes aqueous humor circulation and thus prevents iridectomy. A large number of myopia patients have iridociliary cysts, but the implantation of ICL is not contraindicated in patients with primary iridociliary cysts. Nevertheless, no reports have been published on postoperative EVO-ICL decentration and tilt. Thus, careful observations were required to monitor any possible adverse effects on the visual acuity post-EVO-ICL implantation.

According to our results, good CDVA and UDVA were achieved in all patients, which were equal to or higher than their preoperative levels with no complications. Therefore, primary iridociliary cysts exerted no direct effects on visual performance after the surgical procedures of ICL implantation, which is consistent with previous findings [8, 9]. Our present findings show the safety and efficacy of EVO-ICL implants applied to treat ametropia with iridociliary cysts.

In the preoperative and postoperative IOP values as well as those established at the 1-, 6-, and 12-month intervals, no statistical differences were noted within or between groups. Our results regarding IOP are in line with previous research [8–11]. The postoperative values of ACA, ACV, and ACD were statistically significantly lower than the preoperative in both groups. Nevertheless, no statistical differences were observed between groups regarding ACA, ACV, and ACD. Our findings indicated that the IOP, ACA, ACV, and ACD values in both groups, determined at the 1-, 6-, and 12-month follow-up examination, were relatively stable. This was in agreement with an earlier report by Fernandez-Vigo et al. [12]^,^ who detected ACA narrowing one month postoperatively. Still, ACA remained stable until the two-year follow-up examination. Elevated IOP was found to occur most frequently on the first postoperative day due to retained viscoelastic material; long-term IOP elevation was established as related mainly to the angle closure and pupillary block [13]. ICL implantation pushed the iris forward, causing a reduction in ACA. However, IOP was normal, indicating that although the anterior chamber was changed, the circulation of the aqueous humor was not affected. Notably, an additional 360 *μ*m-diameter hole was designed in the EVO-ICL, which was located in the optical center. That hole was created to facilitate the penetration of the aqueous humor, which reduced the risk of a postICL implantation IOP elevation. Our results showed that the postoperative morphological and structural changes of the anterior segment were stable in both the groups. The presence of cysts and the changes in the ACA did not cause an increase in IOP. We established stable postoperative values of IOP, ACA, ACV, and ACD within the normal range.

We observed the vaults in the cyst group and the noncyst group at 1, 6, and 12 months postoperatively to assess the effect of cysts on ICL position. We found no statistically significant between-group differences. Our study revealed that the presence of cysts exerted no direct impact on the postoperative vaults and the ICL haptic, which may be related to the cysts' peripheral location and relatively small size. Aman-Ullah et al. [14] described a case of binocular iris cysts with TICL implants, which remained stable over 15 months of follow-up examination. Moreover, the authors found that the isolated temporal iris cysts did not influence the TICL location or the final visual outcome. Here, we established a continuous vault value decrease over time, but the vaults of the ICLs after surgery were within a normal range in all patients. Alfonso et al. [15] showed that a larger than 20 *μ*m monthly decline occurred in the vaults during six months postoperatively; then, this decrease was reduced to approximately 2 *μ*m a month at the 36-month follow-up examination. In another study, Chen et al. [16] followed up 270 eyes of 135 patients with iris cysts for one year postICL implantation and found that the changes in the ICL vault were less than 30 *μ*m. In the present study, we selected ICL based on WTW, ACD, crystalline lens rise, diopter, pupil size, and age. After surgery, the values of the ICL vaults were from 200 to 890 *μ*m, with no significant change, showing no influence exerted by the presence of the cyst on that parameter. Additionally, Zeng et al. [7] revealed that many factors, including WTW, STS, ACD, and ICL size, affected the arch vaults after ICL implantation. The leading causes of the abnormal vaults were age, lens thickness, WTW measurement error, and the presence of a ciliary body cyst. In our work, we carefully considered preoperatively all these factors, which contributed to an excellent postoperative outcome.

The position of the ICL is of great importance since it may affect CDVA and facilitate the postoperative occurrence of complications such as glaucoma and cataracts [11, 17]. A primary iridociliary cyst, particularly in the ciliary sulcus, may prevent the ICL haptic from stretching. Alternatively, the ICL haptic may not be on the same height, causing the ICL to be decentered and tilted. It is possible for persistent cyst growth in the ciliary sulcus to cause displacement of the ICL, thereby increasing its tilt. In refractive surgery, accurate centration is considered to be critical for achieving optimal visual outcomes [18–20]. An alignment should exist between the corrected center position and the optical axis or the corneal anterior surface and the optical axis intersection point [21]. He et al. [22] suggested that an evaluation of the ICL position was feasible based on its central-hole relation to the corneal center. In this respect, based on the visual axis, Holladay et al. [23] proposed a standardized approach for intraocular lenses (IOLs) tilt and decentration measurements, which was associated with CTA or corneal light reflex axis. Zhang et al. [24] suggested that IOL displacement measurements related to CTA were the most appropriate postoperative approach for the evaluation of visual acuity. Accordingly, we assessed the ICL decentration and tilt relative to the CTA. Nevertheless, it was difficult to determine the center location of conventional ICLs like V4 with a Pentacam [12, 25]. Notably, the EVO-ICL featured a central hole, whose inner surface in the Scheimpflug image had a strong reflection. Therefore, we were able to observe ICL directly in its central location in the posterior chamber with the use of a Pentacam. Hence, in the current study, we had the opportunity to directly evaluate the ICL decentration in the posterior chamber according to the center hole position in the Scheimpflug images. Such a procedure is noninvasive, noncontact, convenient, and objective; it also has good reliability and repeatability. At 1, 6, and 12 months postoperatively, the decentration and tilt of ICL were quantitatively measured with a rotating Scheimpflug camera. We found no statistically significant between-group difference. Our results showed that the range of decentration was from 0.03 to 0.66 mm, while the degree of tilt was from 0.22° to 6.27°. The position of the ICL in all patients was relatively stable after the surgery. Our study revealed no effect was exerted by iris cysts on the ICL decentration and tilt.

The influence of the central-hole position on clinical visual indicators was assessed in two earlier studies [26, 27]. In one of these investigations, Pérez-Vives et al. [26] conducted an experiment with a visual simulator; they observed no influence of the three predetermined hole locations on the visual acuity. In the second of the aforementioned studies, Park et al. [27] evaluated the degrees of the decentration of ICL (i.e., within a hole diameter of 1, 2, or 3 from the pupil center) in three groups and found no statistical difference between them. In our study, the degree of ICL decentration and tilt and the CDVA were not statistically significantly correlated. The small sample size used in the present study was its major limitation. Nonetheless, the values of the coefficients of the correlation between the ICL tilt and decentration and CDVA were insignificant. Thus, a correlation between the ICL tilt and decentration and the value of CDVA was nearly absent. Although the present research findings indicated that the ICL decentration and tilt had no effect on the final visual outcome, some studies showed that the EVO-ICL central-hole location could influence the postoperative glare and quality of life. Martínez-Plaza et al. [28] evaluated the effects of the specific EVO-ICL central-hole position on vision quality. They showed that the quality of life, as measured by a pertinent questionnaire, was worse when the central hole had a higher position on the vertical axis and the presence of upward decentration of the central hole, which was established by using the visual axis as a reference basis. The decentration of the ICL hole can lead to bothersome glare and eventually adversely affect the patient's quality of life. Eppig et al. [29] concluded that the presence of an ICL's center hole might result in the perception of ghost images (positive dysphotopsia) and stray light despite the absence on-axis of such effects. In an experimental study conducted by Eom et al. [30], ring and arc images were generated by the ICL hole; the cause for this effect was the light refracted from the central hole's inner surface. It is possible that the ring dysphotopsia could have been associated with arc image merging, which was induced by oblique light. Therefore, the increase in the angle of incidence of incoming light induced higher stray light radiant power. It is noteworthy that this phenomenon might have critical importance in various glare patterns. Considering the possible major influence on the patient-perceived quality of life, in spite of the excellent visual outcomes of the central-hole ICL implantation, we strongly advise that patients diagnosed with cysts should be meticulously examined preoperatively and be informed of the potential risks. For instance, a risk could be posed by an alteration in the size of a cyst; moreover, the ICL haptics might be situated in close proximity or even exactly on a cyst position. In this case, the iris root could be pressed forward, causing ACA closure or narrowing. Furthermore, the ICL haptics might be more likely to break a capsule of a cyst, whose leaking out fluid might cause a severe inflammatory process. Therefore, its preTICL surgery design should be appropriate, and the degrees of rotation, as well as the direction, should be selected and developed in such a way as to avoid the intraimplantation connection between cysts and the haptics. All postoperative changes in IOP, ACA, and visual outcomes should be monitored during an extended follow-up period.

## 5. Conclusion

In summary, the implantation of EVO-ICL in myopic patients suffering from primary iris ciliary body cysts is an effective and safe treatment. Moreover, certain degrees of postoperative ICL decentration and tilt have no adverse effects on visual acuity. Several limitations of the present study should be acknowledged, including its brief follow-up duration, limited sample size, and a small cyst-affected area; additionally, this investigation was a retrospective cohort study, and UBM was not used for the detection of the size and location changes in the iris ciliary body cysts. Moreover, the visual quality and the quality of life were not checked. Therefore, further research studies are required to confirm our present findings.

## Figures and Tables

**Table 1:** Baseline preoperative and demographic indicators of the patients.

Parameters	Cyst group (*N* = 47)		Noncyst group (*N* = 44)		*P* value
	Mean ± SD	Range	Mean ± SD	Range	
Eyes (*n*)	47		44		
Patients (*n*)	26		22		
Sex (female: male)	33:14		32:12		0.82
Age (years)	26.31 ± 5.05	18–37	26.50 ± 6.48	18–39	0.91
Spherical refraction (*D*)	−9.57 ± 2.58	−4.75–16.00	−9.25 ± 2.76	−4.75–18.00	0.56
Cylinder refraction (*D*)	−1.22 ± 0.81	0.00–−3.00	−1.35 ± 0.89	0.00–3.50	0.47
Manifest spherical equivalent (*D*)	−10.18 ± 2.67	−5.25–17.50	−9.92 ± 2.83	−4.75–18.00	0.65
CDVA (log MAR)	0.03 ± 0.06	−0.10–0.20	0.01 ± 0.03	0.00–0.15	0.12
UDVA (log MAR)	1.44 ± 0.24	0.90–2.00	1.37 ± 0.30	0.80–2.00	0.23
IOP (mmHg)	15.21 ± 1.97	10.01–19.30	15.35 ± 3.10	9.40–20.0	0.79
AL (mm)	27.62 ± 1.22	24.89–29.72	27.50 ± 1.29	24.16–30.68	0.64
ACA (°)	41.71 ± 4.62	30.50–51.80	40.51 ± 4.79	30.00–51.70	0.23
ACV (mm^3^)	205.70 ± 29.73	143.00–272.00	208.50 ± 38.45	143.00–262.00	0.69
ACD (mm)	3.26 ± 0.21	2.82–3.82	3.27 ± 0.25	2.80–3.58	0.94
WTW (mm)	11.53 ± 0.29	11.09–12.70	11.54 ± 0.38	10.78–12.34	0.90
CCT (*μ*m)	514.90 ± 30.54	457.00–562.00	519.90 ± 24.32	463.00–568.00	0.39
ECD (cells/mm^2^)	3048 ± 344.3	2211–3717	3049 ± 260.9	2512–3522	0.99

**Table 2:** Simple correlation coefficients for the degrees of the ICL decentration, tilt, and CDVA.

Parameters	Time (mo)	Decentration (mm)		Tilt (°)	
		*r*	*P* value	*r*	*P* value
Cyst group	1	0.175	0.238	0.226	0.127
	6	0.131	0.382	0.135	0.367
	12	0.108	0.468	0.165	0.269

Noncyst group	1	0.032	0.837	0.157	0.308
	6	0.105	0.496	0.227	0.139
	12	0.132	0.394	0.166	0.282

## Data Availability

The datasets used and/or analysed during the current study are available from the corresponding author on reasonable request.
